# Homologous recombination deficiency-driven genomic instability in ovarian cancer as an indicator of *BRCA1* and *BRCA2* variant pathogenicity

**DOI:** 10.1016/j.ajhg.2026.05.015

**Published:** 2026-06-23

**Authors:** Simon Schnaiter, Frédéric R. Santer, Katalin A. Csanaky, Louise Adel Jensen, Alain G. Zeimet, Kyriaki Michailidou, Amanda B. Spurdle, Mads Thomassen

**Affiliations:** 1 Institute of Human Genetics, Medical University of Innsbruck, Innsbruck, Austria; 2 Department of Gynecology and Obstetrics, Medical University of Innsbruck, Innsbruck, Austria; 3 Department of Clinical Genetics, Odense University Hospital, Odense, Denmark; 4 Biostatistics Unit, The Cyprus Institute of Neurology & Genetics, Nicosia, Cyprus; 5 Faculty of Medicine, The University of Queensland, Herston, QLD, Australia

**Keywords:** variant classification, BRCA, HRD, genomic instability, VUS, ovarian cancer, cancer risk

## Abstract

Homologous recombination deficiency (HRD) drives oncogenesis and therapeutic vulnerability in high-grade ovarian cancer (HGOC). While HRD testing is widely used to guide platinum- and especially poly(ADP-ribose) polymerase (PARP)-inhibitor-based therapies, its potential to provide evidence for *BRCA1* and *BRCA2* variant classification under the American College of Medical Genetics and Genomics and the Association for Molecular Pathology (ACMG/AMP) framework has not been assessed. In this Evidence-based Network for the Interpretation of Germline Mutant Alleles (ENIGMA) consortium project, a literature review identified four independent HGOC cohorts reporting details on both *BRCA1* and *BRCA2* pathogenic variant (BRCA^pv^) status and HRD-related genomic instability scores (HRD-GISs), all determined by the Myriad MyChoice HRD+ CDx assay. Likelihood ratios (LRs) were calculated to assess the probability that a *BRCA1* or *BRCA2* variant is pathogenic with a given HRD-GIS^high^ (≥42) or HRD-GIS^low^ (<42) tumor status, and these LRs were mapped to ACMG/AMP evidence strength categories defined by Bayesian modeling. Across the pooled dataset of 4,943 tumors (765 *BRCA*^*pv*^ and 4,178 *BRCA1* or *BRCA2* wild type [BRCA^wt^]), 91.0% of BRCA^pv^ and 30.0% of BRCA^wt^ tumors were HRD-GIS^high^. The LR for a *BRCA1* or *BRCA2* variant being pathogenic in an HRD-GIS^high^ HGOC was 3.03 (95% confidence interval [CI]: 2.88–3.19), corresponding to supporting pathogenic evidence. Conversely, the pooled LR for a variant being pathogenic in an HRD-GIS^low^ HGOC was 0.13 (95% CI: 0.10–0.16), corresponding to moderate benign evidence. The HGOC HRD-GIS determined by the MyChoice HRD+ CDx assay provides statistically robust evidence for *BRCA1* and *BRCA2* variant interpretation. This approach may refine variant classification, particularly for variants of uncertain significance, and enhance clinical decision-making in hereditary and tumor-based cancer risk assessment.

## Teaser

Genomic instability scores for HRD in high-grade ovarian cancer provide robust evidence for *BRCA1* and *BRCA2* variant classification according to ACMG/AMP criteria. Analysis of 4,943 tumors resulted in an evidence strengh of *supporting pathogenic* for HRD-GIS^high^ and *moderate benign* for HRD-GIS^low^, highlighting the clinical utility of genomic instability testing for refining *BRCA1* and *BRCA2* variant interpretation.

## Main text

Homologous recombination deficiency (HRD) is a central driver of oncogenesis, progression, and metastasis in high-grade ovarian cancer (HGOC) and thus has been identified as a critical therapeutic target.^1^^,^^2^ It has been shown that individuals with ovarian cancer harboring *BRCA1* (MIM: 113705) or *BRCA2* (MIM: 600185) pathogenic variants (BRCA^pv^) show an overall survival benefit upon maintenance treatment with poly(ADP-ribose) polymerase inhibitors (PARPis; olaparib) in comparison to individuals with BRCA^wt^ ovarian cancer.^3^ Along the line, the subsequent PAOLA-1 trial proved that not only individuals with BRCA^pv^ ovarian cancer but also individuals with *BRCA2* wild type (BRCA^wt^) HRD-related genomic instability (GI) score (HRD-GIS)^high^ ovarian cancer had a benefit from maintenance therapy with a PARPi (olaparib) in combination with bevacizumab.^4^^,^^5^ Tumor HRD status can be inferred through identification of germline and/or somatic pathogenic variants in homologous recombination repair (HRR) genes and/or assessed by examining patterns of copy-number alterations (CNAs) and loss of heterozygosity (LOH) that are characteristic for GI caused by HRD (HRD-GI).^6^^,^^7^ The presence of HRD-GI predicts therapeutic response and prognosis in HGOC and other cancer entities,^1^^,^^8^^,^^9^^,^^10^ although recent data challenge the predictive value of HRD-GI alone.^11^ Commercial assays (e.g., Myriad MyChoice and Foundation Medicine) and integrated in-house testing kits (e.g., the Illumina TruSight Oncology 500 DNA Kit and SOPHiA DDM HRD Solution) are broadly applied for clinical testing of HRD in ovarian cancer, including HRD-GI, accompanied by locally developed multifactorial analyses for research and diagnostics in ovarian cancer (e.g., the Leuven HRD test,^12^ Scarface Score,^8^ and PIGIS^13^). To our knowledge, the most widely used assays are the Myriad MyChoice HRD+ CDx assay and the Illumina TruSight Oncology 500 DNA Kit, which use the same or a very similar HRD quantification algorithm. The Myriad MyChoice HRD+ CDx assay test is based on targeted sequencing of more than 50,000 SNPs in the genome, and a composite HRD score is calculated, including quantification of LOH regions,^6^ number of allelic imbalance extending to telomeres (telomeric allelic imbalance, TAI),^7^ and large-scale state transitions (LSTs).^14^ The three individual scores are added, resulting in a total HRDscore with a cutoff for positivity of 42.

Beyond these genomic scar metrics, additional genomic features have been explored for HRD assessment, including mutational signatures such as SBS3, characteristic insertion/deletion patterns, and copy-number signatures associated with HRD. Integrated approaches such as HRDetect combine multiple genomic features and may capture complementary aspects of HRD beyond genomic scars alone.

Independent of the molecular pathological analysis of tumors to optimize treatment strategies, genetic testing for germline variants in several HRR-associated genes is performed to identify individuals at increased risk of developing cancer. In particular, *BRCA1* and *BRCA2* are investigated because pathogenic variants in these genes are associated with a higher risk of developing breast and ovarian cancer (*BRCA1*- and *BRCA2*-associated hereditary breast and ovarian cancer [HBOC] [MIM: 604370 and MIM: 612555]), as well as other malignancies.^15^^,^^16^ The American College of Medical Genetics and Genomics and the Association for Molecular Pathology (ACMG/AMP) guidelines are now commonly used to classify identified variants as “benign,” “likely benign,” “variant of uncertain significance (VUS),” “likely pathogenic,” or “pathogenic.”^17^^,^^18^ The ACMG/AMP guidelines, as originally published, were designed for general application to variants and provide a set of weighted parameters that can be applied as considered relevant to any disease-causing gene.

The ClinGen consortium^19^ oversees the establishment and activities of variant curation expert panels (VCEPs) that develop gene-centric specifications of the ACMG/AMP guidelines. The Evidence-based Network for the Interpretation of Germline Mutant Alleles (ENIGMA) consortium^20^ was initiated to develop and apply methods for classifying variants in breast and ovarian cancer predisposition genes and has been instrumental in introducing the use of Bayesian quantitative frameworks to apply a range of different evidence types for cancer gene variant classification.^21^^,^^22^^,^^23^ This research was incorporated into the activities of the ClinGen ENIGMA BRCA1 and BRCA2 VCEPs*,* which generated specifications that elaborated the applicability of the ACMG/AMP criteria as originally published^17^ and also extended the data types that can be considered for classification of variants in these genes.^23^ This included allowance for including evidence toward or against pathogenicity for any independent data type based on likelihood ratios (LRs) derived by clinical calibration; to date, the VCEP has applied LR-based weights drawn from research studies assessing the predictive capacity of breast or variant tumor histopathologic features,^24^^,^^25^ family history information,^26^^,^^27^ and age-stratified presentation in case-control cohorts.^28^^,^^29^

Despite these efforts and continuous advances in molecular diagnostics (e.g., Sahu et al.^30^ and Huang et al.^31^), a large proportion of *BRCA1* or *BRCA2* variants remain VUSs after curation. For example, 44% of the more than 30,000 *BRCA1* and *BRCA2* variants in ClinVar,^32^ with at least a one-star classification, are classified as VUSs. Similar findings were reported in a recent Danish cohort analysis, in which 34% of all identified *BRCA1* or *BRCA2* variants were VUSs.^33^ VUSs are variants for which existing evidence is insufficient to determine their clinical impact. Consequently, neither the presence nor the absence of a VUS in a tested proband allows informed decision-making regarding treatment or preventive measures for the tested VUS-positive proband or their family members.

Here, we assessed the HRD-GIS observed in ovarian cancers as a predictor of *BRCA1* and *BRCA2* variant pathogenicity from four independent, previously published cohorts, with the aim of providing a new parameter applicable within the ClinGen ENIGMA *BRCA1* and *BRCA2* VCEP specifications.

Four independent cohorts were identified in a PubMed literature search using the following keywords: “ovarian cancer,” “HRD,” “real-world evidence (RWE),” or by screening clinical trials testing PARPis (Table 1). Cohorts were included when the following clinical information was reported: (1) histologically confirmed ovarian cancer, (2) BRCA^pv^ or BRCA^wt^ status, and (3) HRD-GI status. Furthermore, only cohorts in which the HRD-GI assessment method was supported by at least two independent cohorts were included. This was only the case for the FDA-approved method, MyChoiceDX. Many other studies were identified but could not be included in the analysis because numbers of BRCA^pv^ GIS^low^ tumors were not specified separately as required for LR calculation but were instead presented jointly with BRCA^pv^ GIS^high^ tumors under an HRD-positive label. Furthermore, the available data did not allow calculating LRs for *BRCA1* and *BRCA2* separately. In three out of four cohorts, it was not differentiated whether the variants were germline or somatic, while one cohort (NOVA) consisted exclusively of samples with somatic variants.

**Table 1:** Cohorts selected for analysis

**Cohort name**	**Type of cohort**	**Initial study goal**	**Histologic type**	**No. of samples**	**GIS method**	**Reference**	**Comments**
Marburg	RWE	optimization of sample preparation from FFPE	mixed, predominantly HGOC	2,374	MyChoice HRD+ CDx	Romey et al.^34^	no detailed histology information
NHS	RWE	survival in routine care	FIGO III/IV, high-grade ovarian, fallopian tube, peritoneal cancer	2,211	MyChoice HRD+ CDx	Morgan et al.^35^	no detailed histology information
Study 19	phase 2 trial	Study 19: olaparib maintenance	HGSOC	68	MyChoice HRD+ CDx	Lheureux et al.^36^	selected for platinum sensitivity
NOVA	phase 3 trial	NOVA: niraparib maintenance	HGOC	290	MyChoice HRD+ CDx	Mirza et al.^37^^,^^38^	somatic PVs only

RWE, real-world evidence; FFPE, formalin-fixed paraffin-embedded; HGSOC, high-grade serous ovarian cancer; HGOC, high-grade ovarian cancer; PVs, pathogenic variants.

The Marburg cohort^34^ was initially collected to validate sample processing for HRD testing and consisted of 2,374 samples with available GISs and *BRCA*^*pv*^ status. The most frequent histology type was high-grade serous ovarian cancer (HGSOC; 86.9%), followed by endometrioid carcinoma (2.8%), clear cell carcinoma (2.4%), other ovarian (2.2%), low-grade SOC (2.0%), and other non-ovary cancers (3.6%). 22 (0.9%) samples were removed from the analysis because they lacked GISs. The available information did not allow the attribution of data on BRCA^pv^ and GISs to individuals stratified by histological subtype. Due to a lack of alternatives, all samples were included in the analysis regardless of histological subtype.

The NHS cohort^35^ was originally analyzed to assess the impact of HRD testing in clinical routine. The NHS cohort consists of 2,211 samples with available GISs and *BRCA*^*pv*^ status. Samples were from individuals newly diagnosed with International Federation of Gynecology and Obstetrics (FIGO) stage III/IV high-grade epithelial ovarian, fallopian tube, or primary peritoneal cancer.

The Study 19 cohort^36^ was initially designed as a phase 2 trial to assess olaparib maintenance in individuals with platinum-sensitive recurrent HGSOC. Data for our analysis were taken from a published long-term response analysis performed on the cohort. Samples were from individuals with HGOC, with no further specification of histological type.

The NOVA cohort^37^ was initially designed as a phase 2 trial to assess niraparib maintenance in individuals with recurrent ovarian cancer without germline BRCA^pv^. Data for our analysis were taken from a published post hoc biomarker analysis of the cohort that included analysis for somatic BRCA^pv^.^38^ Samples were from individuals with predominantly HGOC. Detailed histology types are not available.

**Figure 1:**
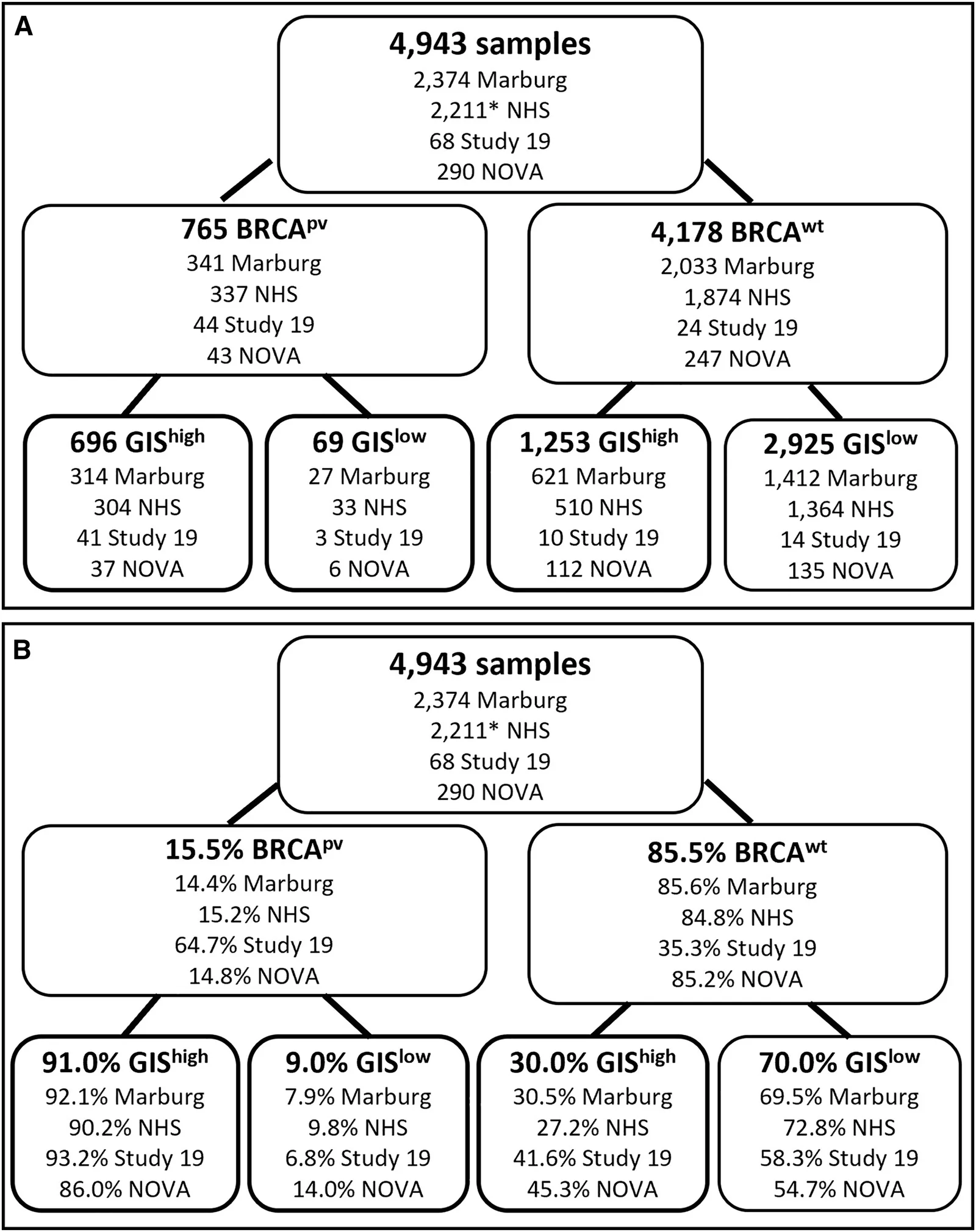
Cohort details Display of distribution of absolute (A) and relative (B) patient numbers in the overall analysis dataset and sub-cohorts. Rectangles outlined in bold mark samples that are clinically categorized as HRD positive due to GIS^high^, irrespective of BRCA^PV^ status. The NHS cohort is marked with an asterisk to highlight that 33 BRCA^pv^-GIS^low^ samples were declared GIS^low^ in the underlying publication^36^ but not enclosed in the total number of samples with successful GIS testing (*n* = 2,178). We added these as successfully tested and GIS^low^ to the cohort for our analysis, resulting in a sample size of 2,211 for this cohort.

In total, 4,943 samples were included in our analysis, of which 765 (15.5%) harbored a BRCA^pv^: 696 (91.0%) were GIS^high^ and 69 (9.0%) were GIS^low^. Of the BRCA^wt^ samples (*n* = 4,178; 84.5%), 1,253 (30.0%) were GIS^high^ and 2,925 (70.0%) were reported as GIS^low^ (Figure 1). Detailed information on the composition of the individual cohorts is depicted in Figure 1.

Loss of BRCA1 or BRCA2 protein function leads to HRD-GI in tumors,^39^ and according to this paradigm, BRCA^pv^ GIS^low^ tumors are unlikely from a molecular biology perspective. Yet, in the analyzed cohorts, 9% (6.8%–14.0%) of the BRCA^pv^ tumors were GIS^low^. This can largely be explained by the specifications of the MyChoiceHRD+ CDx assay, namely a binary threshold of GIS ≥ 42 is required for categorization as HRD-GI positive. No additional detail about individual-level GISs was provided for the BRCA^pv^ GIS^low^ tumors from NHS (33/337, 9.8%) and Study 19 (3/44, 6.8%). However, in the Marburg study,^34^ the authors stated that 10 of 27 BRCA^pv^ GIS^low^ tumors had HRD-GISs within 6 units of the 42 threshold. In the NOVA study,^38^ all 6 BRCA^pv^ GIS^low^ tumors (14.0%) had HRD-GISs greater than 33. These tumors with a GIS of at least 33 display features of HRD-GI but fail to reach the clinical threshold of 42 set for the MyChoice HRD+ CDx test. Since this clinical threshold was defined as the score that encompasses 95% of tumors with *BRCA1* or *BRCA2* pathogenic variants,^9^ it is expected to categorize 5% of BRCA^pv^ tumors as GIS^low^. In the clinical application for identifying responders to PARPi treatment, this does not affect treatment choice, as BRCA^pv^ GIS^low^ tumors are classified as HRD-positive anyway. However, in the context of *BRCA1* and *BRCA2* variant classification, a higher classification strength might be obtained if the cutoff were adjusted or, even better, calibrated using multiple GIS categories (e.g., high, medium, and low).

Another possible explanation for the observation of BRCA^pv^ GIS^low^ test findings within the Marburg cohort is that this was the only cohort that contained ovarian cancer not limited to high-grade cancers. Moreover, four of the BRCA^pv^ GIS^low^ tumors were reported to be of endometrioid histology, in which a *BRCA1* or *BRCA2* variant may represent a germline bystander pathogenic variant not involved in carcinogenesis. Importantly, none of the studies provided conclusive data on “second hits” that would enable differentiation between bystander pathogenic variants and those causally involved. It has been shown that tumors harboring BRCA^pv^ without a second hit exhibit lower HRD-GISs.^40^^,^^41^ However, second-hit information is usually not included in molecular pathology reports, so evidence strengths calibrated without second-hit information are important for variant interpretation in a clinical setting.

The Marburg, NHS, and NOVA cohorts had a very similar distribution of BRCA^pv^ vs. BRCA^wt^, while the share of BRCA^pv^ is much higher in the Study 19 cohort. This may be explained by the Study 19’s trial protocol, which included only individuals responding to platinum-based therapy, thereby favoring BRCA^pv^ and HRD-GI tumors. However, this should not affect the estimation of the LRs, which depend on the ratios of GIS^high^ vs. GIS^low^ within the subgroups of BRCA^pv^ and BRCA^wt^.

Statistical analyses were performed to calculate the relevant LRs for tumor-derived information, as detailed previously.^25^ Tumors of a given cohort were grouped into BRCA^pv^ and BRCA^wt^. Subsequently, each group was divided according to their GIS as determined by the Myriad MyChoice HRD+ CDx assay, leading to subdivision into GIS^high^ (GIS ≥ 42) and GIS^low^ (GIS < 42) (Figure 1A). LRs were calculated for both HRD-GIS groups by comparing their frequency between BRCA^pv^ and BRCA^wt^ as follows:

$$LR=\frac{P\left(pv\right)}{P\left(0\right)}\text{,}$$

where $P\left(pv\right)=\frac{{BRCA}^{pv}\;\left({GIS}^{high}\right)}{{BRCA}^{pv}\;\left({GIS}^{high}\right)+{BRCA}^{pv}\;\left({GIS}^{low}\right)}$ and $P\left(0\right)=\frac{{BRCA}^{wt}\;\left({GIS}^{low}\right)}{{BRCA}^{wt}\;\left({GIS}^{high}\right)+{BRCA}^{wt}\;\left({GIS}^{low}\right)}\text{.}$

The variance of ln(LR) was calculated as previously described by Koopman et al.^42^:

$$Var\left(\ln\left(LR\right)\right)=\frac{1-P\left(pv\right)}{P\left(pv\setminus )\times ({BRCA}^{pv}\;\left({GIS}^{high}\right)+{BRCA}^{pv}\;\left({GIS}^{low}\right)\right)\;}+\frac{1-P\left(0\right)}{P\left(0\right)\times \left({BRCA}^{wt}\left({GIS}^{high}\right)+{BRCA}^{wt}\left({GIS}^{low}\right)\right)\;}\text{.}$$

95% confidence intervals (CIs) were determined to assess the significance of the LR estimates obtained by

$$\text{CI}=e^{\ln\left(LR\right)\;\pm \;1.96(\sqrt{Var\left(\ln\left(LR\right)\right)}}\text{.}$$

Significant LR estimates, i.e., not spanning 1, suggested nominal significance and potential for use as evidence in variant classification.

For application within the context of ACMG/AMG variant classification guidelines, evidence values according to the Bayesian framework of the ACMG/AMP system^18^ were allocated to the LRs. The evidence strength allocated to the LRs is displayed in Table 2.

**Table 2:** Alignment of LR ranges to evidence strength categories of the ACMG/AMG variant classification guidelines

**Evidence category**	**Pathogenic**	**Benign**
Very strong	LR ≥ 350	LR < 0.00285
Strong	18.70 ≤ LR < 350	0.00285 ≤ LR < 0.053
Moderate	4.33 ≤ LR < 18.70	0.053 ≤ LR < 0.23
Supporting	2.08 ≤ LR < 4.33	0.23 ≤ LR < 0.48
Non-informative	0.48 ≤ LR < 2.08	–

Evidence was considered informative only when CIs were statistically significant (did not include 1). LR values within 0.48 ≤ LR < 2.08 were classified as “no evidence applicable,” i.e., does not meet the minimum LR for supporting evidence for or against pathogenicity.

HRD-GIS positivity was assessed for its utility in predicting *BRCA1* or *BRCA2* variant pathogenicity for each cohort separately and for the four-cohort combination (overall analysis).

**Figure 2:**
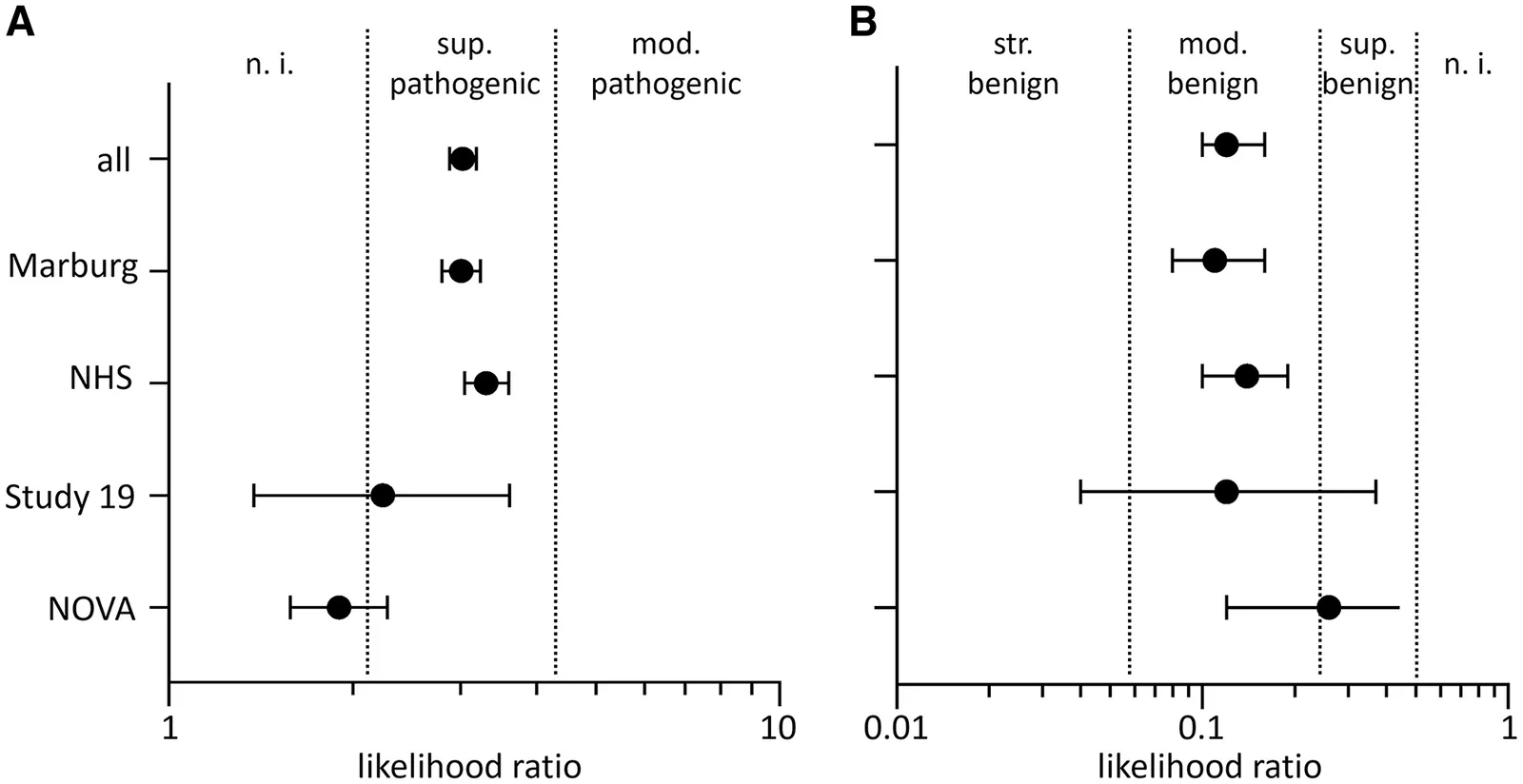
Likelihood ratios with 95% confidence intervals Evidence strengths are indicated in the graphs (n.i., non-informative; sup., supporting; mod., moderate; str., strong) (A) Evidence strength toward pathogenicity based on estimated likelihood ratios (LRs) from the individual and combined studies for a *BRCA1* or *BRCA2* variant in a GIS^high^ HGOC. (B) Evidence strength toward benignity based on estimated LRs from the individual and combined studies for a *BRCA1* or *BRCA2* variant in a GIS^low^ HGOC.

The LR that a given *BRCA1* or *BRCA2* variant in a GIS^high^ HGOC is pathogenic was 3.01 (95% CI: 2.80–3.24) in the Marburg cohort, 3.31 (95% CI: 3.05–3.60) in the NHS cohort, 2.24 (95% CI: 1.38–3.61) in the Study 19 cohort, and 1.90 (95% CI: 1.58–2.28) in the NOVA cohort. The overall LR was 3.03 (95% CI: 2.88–3.19) (Figure 2A). Each of the LRs estimated from three of the individual studies (Marburg, NHS, and Study 19 cohorts), as well as from the pooled analysis, equated to an evidence strength of supporting pathogenic (LR ≥ 2.08–4.30) for a *BRCA1* or *BRCA2* variant found in a GIS^high^ HGOC. LRs estimated from the individual analysis of the NOVA cohort did not equate to any applicable evidence strength.

The LR that a given *BRCA1* or *BRCA2* variant in a GIS^low^ HGOC is pathogenic was estimated to be 0.11 (95% CI: 0.08–0.16) in the Marburg cohort, 0.13 (95% CI: 0.10–0.19) in the NHS cohort, 0.12 (95% CI: 0.04–0.37) in the Study 19 cohort, and 0.26 (95% CI: 0.12–0.54) in the NOVA cohort. The overall LR was calculated to be 0.13 (95% CI: 0.10–0.16) (Figure 2B). Each of the LRs estimated from three of the individual studies (Marburg, NHS, and Study 19 cohorts), as well as from the pooled analysis, equated to an evidence strength of moderate benign (LR ≥ 0.053–0.23) for a *BRCA1* or *BRCA2* variant found in a GIS^low^ HGOC. LR estimated from the individual analysis of the NOVA cohort equated to supporting benign.

The per-cohort results were very similar in three of the four cohorts (Marburg, NHS, and Study 19), with an evidence strength of supporting pathogenic for *BRCA1* or *BRCA2* variants in HRD-GI-positive tumors and moderate benign for *BRCA1* or *BRCA2* variants in HRD-GI-negative tumors. This suggests that our inability to limit the analysis of the Marburg cohort to HGOC tumors, as was achieved in the initial design for the other three cohorts, did not bias our findings. This is unsurprising given that non-HGOC tumors accounted for less than 15% of the Marburg cohort and less than 10% of the overall cohort.

The findings from analysis of the NOVA cohort were less distinctive, providing no evidence of pathogenicity and only supporting evidence against pathogenicity. In contrast to the other three cohorts, which included germline and somatic BRCA^pv^ tumors, the NOVA cohort consists of individuals with exclusively somatic BRCA^pv^, and the samples from the NOVA cohort were taken from recurrent tumor tissue. While further studies will need to clarify whether either of these distinctive characteristics of the cohort caused the reduced evidence strength, this observation demonstrates that limitations have to be considered when applying our results to variant classification.

Another limitation of this study is that only data from predominantly HGOC samples tested using the MyChoice HRD+ CDx assay were available in sufficient quantity and quality for inclusion. As a result, the evidence strengths are only validated for HGOC samples assessed with the MyChoice HRD+ CDx assay. The performance of different algorithms/assays for HRD-GIS measurement varies,^43^ and the applicability of our results is limited to the MyChoice HRD+ CDx assay or HRD-GIS assays thoroughly validated against the Myriad MyChoice HRD+ CDx assay, at the curator’s discretion. Further studies are required to provide evidence-based validation of alternative algorithms/assays and their applicability to other cancer types.

Furthermore, as a literature-based study, it is limited to the data provided by the sources used, which were designed for other purposes than the study in question. Consequently, detailed data, e.g., on second hits in the tumor, variants in other HRD genes (including *BRCA1* promoter methylation), *BRCA1* or *BRCA2* variants of uncertain significance (VUSs), or differentiated data for *BRCA1* and *BRCA2*, which might have allowed more refined results, were not available. Our data indicate that further studies are needed to determine whether our study results are applicable to non-primary tumor samples and individuals with somatic variants.

Our results, albeit with limitations, show that HRD-GI status in HGOC, as assessed by the MyChoice HRD+ CDx assay, is an applicable indicator for interpreting *BRCA1* and *BRCA2* variants and provides significant evidence strength for potential use within ACMG/AMP guideline-based variant interpretation. Our data indicate that supporting pathogenic evidence can be applied to a *BRCA1* or *BRCA2* variant identified in an individual with GIS^high^ HGOC, whereas moderate benign evidence can be applied to a variant identified in an individual with GIS^low^ HGOC. Given that information on HGOC HRD-GI assessed by MyChoice HRD+ CDx is increasingly available because of its value in individual-level therapeutic decision-making, the integration of combined HRD-GI and pathogenic variant status into evidence assessment toward or against pathogenicity may substantially facilitate the routine diagnostic classification of *BRCA1* and *BRCA2* variants and contribute considerably to clarifying the pathogenicity of many variants.

## Data and code availability

This study did not generate datasets or code.

## Web resources

OMIM, https://www.omim.org

## Acknowledgments

L.A.J. was supported by the Shared Research Fund of 10.13039/501100002918Copenhagen University Hospital (Rigshospitalet) and 10.13039/501100004196Odense University Hospital (case no. 123-A5101).

## Author contributions

Conceptualization, S.S., K.A.C., L.A.J., A.B.S., and M.T.; data curation, S.S., K.A.C., and K.M., formal analysis, S.S., K.A.C., and K.M., investigation, S.S., K.A.C., A.G.Z., A.B.S., and M.T.; methodology, S.S., F.R.S., A.B.S., K.M., and M.T.; project administration, S.S., F.R.S., A.B.S., and M.T.; supervision, S.S. and M.T.; validation, S.S. and K.M.; visualization, S.S. and L.A.J.; writing – original draft, S.S., F.R.S., A.B.S., and M.T.; writing – review & editing, S.S., F.R.S., L.A.J., A.G.Z., A.B.S., K.M., and M.T.

## Declaration of interests

S.S. received research funding from AstraZeneca, and A.G.Z. received research funding from AstraZeneca and has had expenses paid by, done consulting for, and is on the advisory board of AstraZeneca, GSK, and Tesaro.

## References

[1] Ngoi N.Y.L., Tan D.S.P. (2021). The role of homologous recombination deficiency testing in ovarian cancer and its clinical implications: do we need it?. ESMO Open.

[2] Stecklein S.R., Jensen R.A. (2012). Identifying and exploiting defects in the Fanconi anemia/BRCA pathway in oncology. Transl. Res..

[3] Moore K., Colombo N., Scambia G., Kim B.-G., Oaknin A., Friedlander M., Lisyanskaya A., Floquet A., Leary A., Sonke G.S. (2018). Maintenance Olaparib in Patients with Newly Diagnosed Advanced Ovarian Cancer. N. Engl. J. Med..

[4] Lord C.J., Ashworth A. (2017). PARP inhibitors: Synthetic lethality in the clinic. Science.

[5] Ray-Coquard I., Pautier P., Pignata S., Pérol D., González-Martín A., Berger R., Fujiwara K., Vergote I., Colombo N., Mäenpää J. (2019). Olaparib plus Bevacizumab as First-Line Maintenance in Ovarian Cancer. N. Engl. J. Med..

[6] Abkevich V., Timms K.M., Hennessy B.T., Potter J., Carey M.S., Meyer L.A., Smith-McCune K., Broaddus R., Lu K.H., Chen J. (2012). Patterns of genomic loss of heterozygosity predict homologous recombination repair defects in epithelial ovarian cancer. Br. J. Cancer.

[7] Birkbak N.J., Wang Z.C., Kim J.-Y., Eklund A.C., Li Q., Tian R., Bowman-Colin C., Li Y., Greene-Colozzi A., Iglehart J.D. (2012). Telomeric allelic imbalance indicates defective DNA repair and sensitivity to DNA-damaging agents. Cancer Discov..

[8] Fernández-Serra A., López-Reig R., Márquez R., Gallego A., de Sande L.M., Yubero A., Pérez-Segura C., Ramchandani-Vaswani A., Barretina-Ginesta M.P., Mendizábal E. (2023). The Scarface Score: Deciphering Response to DNA Damage Agents in High-Grade Serous Ovarian Cancer-A GEICO Study. Cancers (Basel).

[9] Telli M.L., Timms K.M., Reid J., Hennessy B., Mills G.B., Jensen K.C., Szallasi Z., Barry W.T., Winer E.P., Tung N.M. (2016). Homologous Recombination Deficiency (HRD) Score Predicts Response to Platinum-Containing Neoadjuvant Chemotherapy in Patients with Triple-Negative Breast Cancer. Clin. Cancer Res..

[10] Ledermann J.A., Drew Y., Kristeleit R.S. (2016). Homologous recombination deficiency and ovarian cancer. Eur. J. Cancer.

[11] Fiegl H., Schnaiter S., Reimer D.U., Leitner K., Nardelli P., Tsibulak I., Wieser V., Wimmer K., Schamschula E., Marth C., Zeimet A.G. (2024). BRCA loss of function including BRCA1 DNA-methylation, but not BRCA-unrelated homologous recombination deficiency, is associated with platinum hypersensitivity in high-grade ovarian cancer. Clin. Epigenetics.

[12] Loverix L., Vergote I., Busschaert P., Vanderstichele A., Venken T., Boeckx B., Harter P., Brems H., Van Nieuwenhuysen E., Pignata S. (2023). PARP inhibitor predictive value of the Leuven HRD test compared with Myriad MyChoice CDx PLUS HRD on 468 ovarian cancer patients from the PAOLA-1/ENGOT-ov25 trial. Eur. J. Cancer.

[13] Schnaiter S., Schamschula E., Laschtowiczka J., Fiegl H., Zschocke J., Zeimet A., Wimmer K., Reimer D. (2024). Stratification of Homologous Recombination Deficiency-Negative High-Grade Ovarian Cancer by the Type of Peritoneal Spread into Two Groups with Distinct Survival Outcomes. Cancers (Basel).

[14] Popova T., Manié E., Rieunier G., Caux-Moncoutier V., Tirapo C., Dubois T., Delattre O., Sigal-Zafrani B., Bollet M., Longy M. (2012). Ploidy and large-scale genomic instability consistently identify basal-like breast carcinomas with BRCA1/2 inactivation. Cancer Res..

[15] Antoniou A., Pharoah P.D.P., Narod S., Risch H.A., Eyfjord J.E., Hopper J.L., Loman N., Olsson H., Johannsson O., Borg Å. (2003). Average risks of breast and ovarian cancer associated with BRCA1 or BRCA2 mutations detected in case Series unselected for family history: a combined analysis of 22 studies. Am. J. Hum. Genet..

[16] Petrucelli N., Daly M.B., Pal T., Adam M.P., Feldman J., Mirzaa G.M., Pagon R.A., Wallace S.E., Amemiya A. (1993). GeneReviews®.

[17] Richards S., Aziz N., Bale S., Bick D., Das S., Gastier-Foster J., Grody W.W., Hegde M., Lyon E., Spector E. (2015). Standards and guidelines for the interpretation of sequence variants: a joint consensus recommendation of the American College of Medical Genetics and Genomics and the Association for Molecular Pathology. Genet. Med..

[18] Tavtigian S.V., Greenblatt M.S., Harrison S.M., Nussbaum R.L., Prabhu S.A., Boucher K.M., Biesecker L.G., ClinGen Sequence Variant Interpretation Working Group (ClinGen SVI) (2018). Modeling the ACMG/AMP variant classification guidelines as a Bayesian classification framework. Genet. Med..

[19] Andersen E.F., Azzariti D.R., Babb L., Berg J.S., Biesecker L.G., Bly Z., Buchanan A.H., DiStefano M.T., Gong L., Harrison S.M. (2025). The Clinical Genome Resource (ClinGen): Advancing genomic knowledge through global curation. Genet. Med..

[20] Spurdle A.B., Healey S., Devereau A., Hogervorst F.B.L., Monteiro A.N.A., Nathanson K.L., Radice P., Stoppa-Lyonnet D., Tavtigian S., Wappenschmidt B. (2012). ENIGMA--evidence-based network for the interpretation of germline mutant alleles: an international initiative to evaluate risk and clinical significance associated with sequence variation in BRCA1 and BRCA2 genes. Hum. Mutat..

[21] Plon S.E., Eccles D.M., Easton D., Foulkes W.D., Genuardi M., Greenblatt M.S., Hogervorst F.B.L., Hoogerbrugge N., Spurdle A.B., Tavtigian S.V. (2008). Sequence variant classification and reporting: recommendations for improving the interpretation of cancer susceptibility genetic test results. Hum. Mutat..

[22] Goldgar D.E., Easton D.F., Byrnes G.B., Spurdle A.B., Iversen E.S., Greenblatt M.S., IARC Unclassified Genetic Variants Working Group (2008). Genetic evidence and integration of various data sources for classifying uncertain variants into a single model. Hum. Mutat..

[23] Parsons M.T., Tudini E., Li H., Hahnen E., Wappenschmidt B., Feliubadaló L., Aalfs C.M., Agata S., Aittomäki K., Alducci E. (2019). Large scale multifactorial likelihood quantitative analysis of BRCA1 and BRCA2 variants: An ENIGMA resource to support clinical variant classification. Hum. Mutat..

[24] Spurdle A.B., Couch F.J., Parsons M.T., McGuffog L., Barrowdale D., Bolla M.K., Wang Q., Healey S., Schmutzler R.K., Wappenschmidt B. (2014). Refined histopathological predictors of BRCA1 and BRCA2 mutation status: a large-scale analysis of breast cancer characteristics from the BCAC, CIMBA, and ENIGMA consortia. Breast Cancer Res..

[25] O’Mahony D.G., Ramus S.J., Southey M.C., Meagher N.S., Hadjisavvas A., John E.M., Hamann U., Imyanitov E.N., Andrulis I.L., Sharma P. (2023). Ovarian cancer pathology characteristics as predictors of variant pathogenicity in BRCA1 and BRCA2. Br. J. Cancer.

[26] Easton D.F., Deffenbaugh A.M., Pruss D., Frye C., Wenstrup R.J., Allen-Brady K., Tavtigian S.V., Monteiro A.N.A., Iversen E.S., Couch F.J., Goldgar D.E. (2007). A systematic genetic assessment of 1,433 sequence variants of unknown clinical significance in the BRCA1 and BRCA2 breast cancer-predisposition genes. Am. J. Hum. Genet..

[27] Li H., LaDuca H., Pesaran T., Chao E.C., Dolinsky J.S., Parsons M., Spurdle A.B., Polley E.C., Shimelis H., Hart S.N. (2020). Classification of variants of uncertain significance in BRCA1 and BRCA2 using personal and family history of cancer from individuals in a large hereditary cancer multigene panel testing cohort. Genet. Med..

[28] Zanti M., O’Mahony D.G., Parsons M.T., Li H., Dennis J., Aittomäkkiki K., Andrulis I.L., Anton-Culver H., Aronson K.J., Augustinsson A. (2023). A likelihood ratio approach for utilizing case-control data in the clinical classification of rare sequence variants: application to BRCA1 and BRCA2. Hum. Mutat..

[29] Zanti M., O’Mahony D.G., Parsons M.T., Dorling L., Dennis J., Boddicker N.J., Chen W., Hu C., Naven M., Yiangou K. (2025). Analysis of more than 400,000 women provides case-control evidence for BRCA1 and BRCA2 variant classification. Nat. Commun..

[30] Sahu S., Galloux M., Southon E., Caylor D., Sullivan T., Arnaudi M., Zanti M., Geh J., Chari R., Michailidou K. (2025). Saturation genome editing-based clinical classification of BRCA2 variants. Nature.

[31] Huang H., Hu C., Na J., Hart S.N., Gnanaolivu R.D., Abozaid M., Rao T., Tecleab Y.A., Ambrosone C.B., Yao S. (2025). Functional evaluation and clinical classification of BRCA2 variants. Nature.

[32] Landrum M.J., Kattman B.L. (2018). ClinVar at five years: Delivering on the promise. Hum. Mutat..

[33] Munch A.K., Feldner E.S., Bækgaard C.H., Larsen M.B., Slemming-Adamsen N., Boonen D.S., Møller N.B., Pedersen I.S., Hansen T.V.O., Terkelsen T. (2025). Classification of Gene Variants in a Danish Population with Suspected Predisposition to Hereditary Breast and/or Ovarian Cancer. Cancers (Basel).

[34] Romey M., Rodepeter F., Hattesohl A., Kaiser K., Teply-Szymanski J., Heitz F., Staebler A., Serra V., Grass A., Marmé F. (2024). Systematic Analysis of Homologous Recombination Deficiency Testing in Ovarian Cancer-Development of Recommendations for Optimal Assay Performance. Mod. Pathol..

[35] Morgan R.D., Clamp A.R., Barnes B.M., Timms K., Schlecht H., Yarram-Smith L., Wallis Y., Valganon-Petrizan M., MacMahon S., White R. (2023). Homologous recombination deficiency in newly diagnosed FIGO stage III/IV high-grade epithelial ovarian cancer: a multi-national observational study. Int. J. Gynecol. Cancer.

[36] Lheureux S., Lai Z., Dougherty B.A., Runswick S., Hodgson D.R., Timms K.M., Lanchbury J.S., Kaye S., Gourley C., Bowtell D. (2017). Long-Term Responders on Olaparib Maintenance in High-Grade Serous Ovarian Cancer: Clinical and Molecular Characterization. Clin. Cancer Res..

[37] Mirza M.R., Monk B.J., Herrstedt J., Oza A.M., Mahner S., Redondo A., Fabbro M., Ledermann J.A., Lorusso D., Vergote I. (2016). Niraparib Maintenance Therapy in Platinum-Sensitive, Recurrent Ovarian Cancer. N. Engl. J. Med..

[38] Mirza M.R., Lindahl G., Mahner S., Redondo A., Fabbro M., Rimel B.J., Herrstedt J., Oza A.M., Canzler U., Berek J.S. (2022). Ad hoc Analysis of the Phase III ENGOT-OV16/NOVA Study: Niraparib Efficacy in Germline BRCA Wild-type Recurrent Ovarian Cancer with Homologous Recombination Repair Defects. Cancer Res. Commun..

[39] Farmer H., McCabe N., Lord C.J., Tutt A.N.J., Johnson D.A., Richardson T.B., Santarosa M., Dillon K.J., Hickson I., Knights C. (2005). Targeting the DNA repair defect in BRCA mutant cells as a therapeutic strategy. Nature.

[40] Maxwell K.N., Wubbenhorst B., Wenz B.M., De Sloover D., Pluta J., Emery L., Barrett A., Kraya A.A., Anastopoulos I.N., Yu S. (2017). BRCA locus-specific loss of heterozygosity in germline BRCA1 and BRCA2 carriers. Nat. Commun..

[41] Fortuno C., Zhang J., Koufariotis L.T., Hollway G., Wood S., Pearson J.V., Simpson P.T., Lakhani S.R., McCart Reed A.E., Thorne H. (2026). Integrating breast tumour homologous recombination deficiency status to aid germline BRCA1 and BRCA2 variant classification. EBioMedicine.

[42] Koopman P.A.R. (1984). Confidence Intervals for the Ratio of Two Binomial Proportions. Biometrics.

[43] Mark L.R., Terp S.K., Krarup H.B., Thomassen M., Pedersen I.S., Bøgsted M. (2023). Homologous Recombination Deficiency Detection Algorithms: A Systematic Review. Cancers (Basel).

